# Data describing the flow-mediated vasodilation responses and blood pressure in young adult humans after a single dose of oral edible emu oil

**DOI:** 10.1016/j.dib.2018.01.015

**Published:** 2018-02-02

**Authors:** Tadayoshi Miyashita, Ryosuke Koizumi, Yoshimasa Sagane, Koichi Niwa, Toshihiro Watanabe, Kazuhiro Minami

**Affiliations:** a DHC Corporation, 2–7-1 Minami-Azabu, Minato-ku, Tokyo 106–8571, Japan; b NODAI Research Institute, 1-1-1 Sakuragaoka, Setagaya-ku, Tokyo 156–8502, Japan; c Department of Food and Cosmetic Science, Faculty of Bioindustry, Tokyo University of Agriculture, 196 Yasaka, Abashiri 099–2493, Japan

## Abstract

The data provided herein include flow-mediated vasodilation responses, represented by changes in arterial diameter, and blood pressure in young adults after a single oral dose of edible emu oil or placebo (cross-over design). Ten healthy men and 10 healthy women participated. Increased blood flow in the antebrachial region was induced by inflating a pressure cuff and subsequently releasing the pressure by deflating the cuff. After the release, the arterial diameter was continuously monitored for 110 sec using ultrasonic diagnostic equipment. The changes in the arterial diameter from 20 to 110 sec post-cuff deflation are described in line graphs and tables. In addition, systolic and diastolic blood pressure data are provided in a table.

**Specifications Table**

**Table:** 

Subject area	*Biology*
More specific subject area	*Vascular physiology*
Type of data	*Tables and Figures*
How data was acquired	*Flow-mediated vasodilation test using ultrasonic diagnostic equipment*
Data format	*Raw, Analyzed*
Experimental factors	*The brachial artery was monitored using ultrasonic diagnostic equipment and the diameter was calculated. In addition, blood pressure was assessed.*
Experimental features	*A cross-over design was utilized. The emu oil/placebo was administered under conditions of dietary restriction to avoid confounding.*
Data source location	*Abashiri, Japan*
Data accessibility	*The data are supplied with this article*

**Value of the data**

• These data are available to determine the effect of emu oil on blood flow-mediated vasodilation responses and blood pressure.
• The data presented here may be used as a reference for comparisons with other foods that have an effect on flow-mediated vasodilation responses and blood pressure.
• This data offers a valuable and searchable resource on functional foods and can be used for future functional food studies.

## 1 Data

**Fig. 1:**
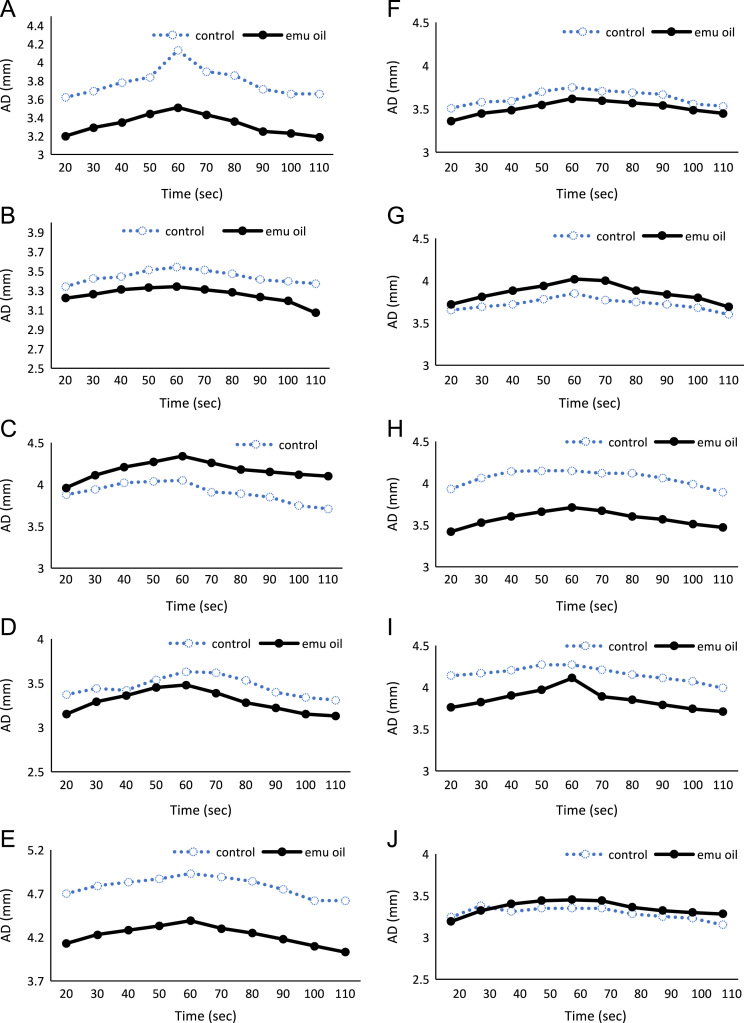
Changes in the arterial diameter in emu oil and placebo dosed male participants after releasing pressure applied to the antebrachial region.

**Fig. 2:**
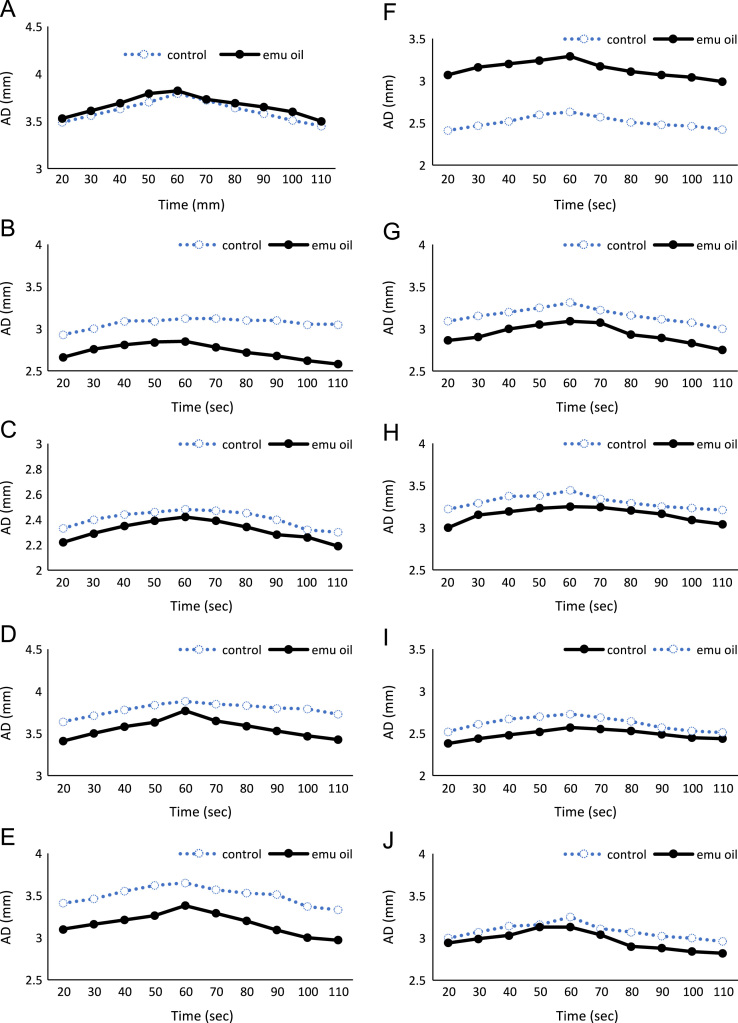
Changes in the arterial diameter in emu oil or placebo dosed female participants after releasing pressure applied to the antebrachial region.

Flow-mediated vasodilation is the dilation response of the artery to the shear stress of blood flow, and has been utilized in clinical medicine and functional food studies as a noninvasive method to examine endothelial function [1]. Flow-mediated vasodilation can be represented by the changes in brachial arterial diameter after increased blood flow. To produce the condition of increased blood-flow, pressure can be applied using a pressure cuff and then released by deflating the cuff [2], [3]. Fig. 1, Fig. 2 show the changes in the arterial diameter after releasing pressure applied to the antebrachial region via a pressure cuff in emu oil or placebo dosed men and women. Table 1 provides the numeric arterial diameter data used to generate the line graphs in Fig. 1. Blood pressure data are provided in Table 2.

**Table 1:** Changes in the arterial diameter (mm) after releasing pressure applied to the antebrachial region in emu oil and placebo dosed participants.

	Participant	Time (sec)	20	30	40	50	60	70	80	90	100	110
Male	A	Placebo	3.62	3.69	3.78	3.84	4.13	3.90	3.86	3.71	3.66	3.66
		Emu oil	3.20	3.29	3.35	3.44	3.51	3.43	3.36	3.25	3.23	3.19
	B	Placebo	3.34	3.42	3.44	3.51	3.54	3.51	3.47	3.41	3.39	3.37
		Emu oil	3.22	3.26	3.31	3.33	3.34	3.31	3.28	3.23	3.19	3.07
	C	Placebo	3.88	3.94	4.02	4.04	4.05	3.91	3.89	3.85	3.75	3.71
		Emu oil	3.96	4.11	4.21	4.27	4.34	4.26	4.18	4.15	4.12	4.10
	D	Placebo	3.37	3.44	3.42	3.54	3.63	3.62	3.53	3.40	3.34	3.31
		Emu oil	3.15	3.29	3.36	3.45	3.48	3.39	3.28	3.22	3.15	3.13
	E	Placebo	4.70	4.79	4.83	4.87	4.93	4.89	4.84	4.75	4.62	4.62
		Emu oil	4.13	4.23	4.28	4.33	4.39	4.30	4.25	4.18	4.10	4.03
	F	Placebo	3.51	3.58	3.59	3.70	3.75	3.71	3.69	3.67	3.56	3.53
		Emu oil	3.36	3.45	3.49	3.55	3.62	3.60	3.57	3.54	3.49	3.45
	G	Placebo	3.65	3.69	3.72	3.78	3.85	3.77	3.75	3.72	3.68	3.60
		Emu oil	3.72	3.81	3.88	3.94	4.02	4.00	3.88	3.84	3.80	3.69
	H	Placebo	3.93	4.06	4.14	4.15	4.15	4.12	4.12	4.06	3.99	3.89
		Emu oil	3.42	3.53	3.60	3.66	3.71	3.67	3.60	3.57	3.51	3.47
	I	Placebo	4.14	4.17	4.20	4.27	4.27	4.21	4.15	4.11	4.07	3.99
		Emu oil	3.76	3.82	3.90	3.97	4.11	3.89	3.85	3.79	3.74	3.71
	J	Placebo	3.24	3.38	3.31	3.35	3.35	3.35	3.28	3.25	3.23	3.15
		Emu oil	3.19	3.32	3.40	3.44	3.45	3.44	3.36	3.32	3.30	3.28
Female	A	Placebo	3.49	3.56	3.63	3.70	3.79	3.72	3.64	3.58	3.51	3.45
		Emu oil	3.53	3.61	3.69	3.79	3.82	3.73	3.69	3.65	3.60	3.50
	B	Placebo	2.93	3.00	3.09	3.09	3.12	3.12	3.10	3.10	3.05	3.05
		Emu oil	2.66	2.76	2.81	2.84	2.85	2.78	2.72	2.68	2.62	2.58
	C	Placebo	2.33	2.40	2.44	2.46	2.48	2.47	2.45	2.40	2.32	2.30
		Emu oil	2.22	2.29	2.35	2.39	2.42	2.39	2.34	2.28	2.26	2.19
	D	Placebo	3.64	3.71	3.78	3.84	3.88	3.85	3.83	3.80	3.79	3.73
		Emu oil	3.41	3.50	3.58	3.63	3.77	3.65	3.59	3.53	3.47	3.43
	E	Placebo	3.41	3.46	3.55	3.62	3.65	3.57	3.53	3.51	3.37	3.33
		Emu oil	3.10	3.16	3.21	3.26	3.38	3.29	3.20	3.09	3.00	2.97
	F	Placebo	2.41	2.47	2.52	2.60	2.63	2.57	2.51	2.48	2.46	2.42
		Emu oil	3.07	3.16	3.20	3.24	3.29	3.17	3.11	3.07	3.04	2.99
	G	Placebo	3.09	3.15	3.20	3.25	3.31	3.22	3.16	3.11	3.07	3.00
		Emu oil	2.86	2.90	3.00	3.05	3.09	3.07	2.93	2.89	2.83	2.75
	H	Placebo	3.22	3.29	3.37	3.38	3.44	3.34	3.29	3.25	3.23	3.21
		Emu oil	3.00	3.15	3.19	3.23	3.25	3.24	3.20	3.16	3.09	3.04
	I	Placebo	2.38	2.44	2.48	2.52	2.57	2.55	2.53	2.49	2.45	2.44
		Emu oil	2.52	2.61	2.67	2.70	2.73	2.69	2.64	2.57	2.53	2.51
	J	Placebo	3.00	3.07	3.14	3.16	3.25	3.11	3.07	3.02	3.00	2.96
		Emu oil	2.94	2.99	3.03	3.13	3.13	3.04	2.90	2.88	2.84	2.82

**Table 2:** Blood pressure in emu oil and placebo dosed participants.

		Blood Pressure (mmHg)			
		Systolic		Diastolic	
	Participant	Placebo	Emu oil	Placebo	Emu oil
Male	A	115	113	72	64
	B	122	125	67	67
	C	121	123	62	65
	D	126	135	56	51
	E	115	117	63	59
	F	116	118	68	67
	G	118	121	65	61
	H	109	110	66	68
	I	110	97	64	54
	J	114	111	56	58
Female	A	111	110	74	75
	B	95	97	56	57
	C	103	101	63	61
	D	111	100	64	59
	E	98	97	55	53
	F	107	103	67	68
	G	98	100	71	69
	H	96	98	63	57
	I	108	112	69	70
	J	122	123	76	72

## 2 Experimental design, materials and methods

### 2.1 Participants

Twenty healthy students (10 males and 10 females; age range, 19–21 years) from the Hokkaido Okhotsk Campus of the Tokyo University of Agriculture were recruited in July 2017, according to the inclusion and exclusion criteria shown in Supplementary Document 1. The study protocol was approved by the Tokyo University of Agriculture Committee of Human Subject Research Ethics. Informed consent was obtained from all participants.

### 2.2 Materials

Edible emu oil, extracted from the subcutaneous fat of the emu (*Dromaius novaehollandiae*), was purchased from Tokyo NODAI Bio-Industry (Abashiri, Japan). Fifty milligrams of the oil were encapsulated into soft gelatin capsules (Matsuya, Osaka, Japan). Dose of emu oil was determined based on the amount in blending quantity in the commercially available emu oil capsule, to avoid the unexpected side effect due to emu oil even though the possible side effect has not found in the oil.

### 2.3 Food restriction protocol and flow-mediated vasodilation measurement

The data were collected at the Hokkaido Okhotsk Campus of the Tokyo University of Agriculture between July and August 2017. Using a cross-over design, each participant was randomly administered a single dose of the emu oil or tap water (placebo) in a soft capsule. The second dose of the capsule, which is another sample from single dose, was administered more than a week later. Participants were asked not to take any nutritional supplements on the day before and the morning of the test, with the exception of 90–130 g/meal of cereal (Flugra, Calbee, Tokyo, Japan) and 600–800 mL/day of milk, to meet Dietary Reference Intakes for Japanese 2015 from Ministry of Health, Labour and Welfere of Japan.

Flow-mediated vasodilation was measured 3 h after the oil was administered. Precise criteria for the measurement based on the Guidelines for non-invasive vascular function test [4] is described in Supplementary document 2. To arrest the blood flow in the brachial artery, pressure (blood pressure plus 40 mmHg) was applied to the antebrachial region for 5 min using the cuff of a mercurial sphygmomanometer (Yamasu, Japan). Avascularization was monitored by using color Doppler option of ultrasonic diagnostic equipment (LOGIQ P6, GE Healthcare, Little Chalfont, UK). The pressure was released by deflating the cuff, and the brachial artery was monitored continuously using LOGIQ P6. Electro-cardiogram was concomitantly monitored to detect diastolic potential. Arterial diameter (AD) when the maximum diastolic potential was measured using the custom-designed edge-detection software FMDscope (Media Cross Co. Ltd, Japan). The AD was determined based on average of 10-times measurements. Potential error in measurements was detected by using trend graph of AD, and omitted from the calculation of average score. Blood pressure was measured using an electronic sphygmomanometer (ES-P2000, Terumo, Tokyo, Japan). We confirmed that no significant side effect was observed from any participant in this study.

## Funding

This research did not receive any specific grant from funding agencies in the public, commercial, or not-for-profit sectors.
