# Supplementary material for: Data describing the flow-mediated vasodilation responses and blood pressure in young adult humans after a single dose of oral edible emu oil
Source: Data Brief. 2018 Feb 2;17:631–7. doi: 10.1016/j.dib.2018.01.015 (PMC5852277; doi:10.1016/j.dib.2018.01.015)
Supplement: Supplementary file 2 — Supplementary material [file mmc2.docx]

**Supplementary document 1**

Participant inclusion and exclusion criteria

Inclusion criteria

1. Generally healthy male or female individual with BMI value between 18.5 to 25.0, inclusive

2. Able to understand the study and to read, understand, and sign an informed consent agreement

3. In case of female, individual in one to seven days in menstrual cycle

Exclusive criteria

1. Individual under medical treatment

2. History of smoking habit

3. Individual with habitual excessive exercises and training

4. History of disease in circulatory system, such as hypertension, diabetes mellitus, or dyslipidemia

5. Postmenopausal female

**Supplementary document 2**

Criteria for flow-mediated vasodilation measurement

1. Temperature in room for the measurement is kept at 25 ˚C.

2. The participant keep at rest in a sitting posture for at least 30 min before measurement

3. Measurement is performed in a sitting posture.

4. All measurements are performed in 10 a.m.

5. Any exercises and smoking are prohibited

6. Meals in a day before measuring and the morning on the day of measuring are unified

7. Any conversation and sleeping during the measurement are prohibited
